# Structure–Activity Relationship Analysis of Flavonoids and Its Inhibitory Activity Against BACE1 Enzyme Toward a Better Therapy for Alzheimer’s Disease

**DOI:** 10.3389/fchem.2022.874615

**Published:** 2022-06-27

**Authors:** Nur Intan Saidaah Mohamed Yusof, Zafirah Liyana Abdullah, Norodiyah Othman, Fazlin Mohd Fauzi

**Affiliations:** ^1^ Faculty of Pharmacy, Universiti Teknologi MARA Selangor, Selangor, Malaysia; ^2^ Haematology Unit, Cancer Research Centre, Institute for Medical Research, National Institutes of Health Complex, Selangor, Malaysia; ^3^ Collaborative Drug Discovery Research, Faculty of Pharmacy, Universiti Teknologi MARA Selangor, Selangor, Malaysia

**Keywords:** flavonoids, Alzheimer’s disease, BACE1, molecular docking, structure–activity relationship (SAR)

## Abstract

Drug development in Alzheimer’s disease (AD) suffers from a high attrition rate. In 2021, 117 agents tested in phases I and II and 36 agents tested in phase III were discontinued. Natural product compounds may be good lead compounds for AD as they contain functional groups that are important for binding against key AD targets such as β-secretase enzyme (BACE1). Hence, in this study, 64 flavonoids collected from rigorous literature search and screening that have been tested from 2010 to 2022 against BACE1, which interferes in the formation of amyloid plaque, were analyzed. The 64 unique flavonoids can be further classified into five core fragments. The flavonoids were subjected to clustering analysis based on its structure, and each representative of the clusters was subjected to molecular docking. There were 12 clusters formed, where only 1 cluster contained compounds from two different core fragments. Several observations can be made where 1) flavanones with sugar moieties showed higher inhibitory activity compared to flavanones without sugar moieties. The number of sugar moieties and position of glycosidic linkage may also affect the inhibitory activity. 2) Non-piperazine-substituted chalcones when substituted with functional groups with decreasing electronegativity at the *para* position of both rings result in a decrease in inhibitory activity. Molecular docking indicates that ring A is involved in hydrogen bond, whereas ring B is involved in van der Waals interaction with BACE1. 3) Hydrogen bond is an important interaction with the catalytic sites of BACE1, which are Asp32 and Asp228. As flavonoids contain favorable structures and properties, this makes them an interesting lead compound for BACE1. However, to date, no flavonoids have made it through clinical trials. Hence, these findings may aid in the design of highly potent and specific BACE1 inhibitors, which could delay the progression of AD.

## Introduction

Alzheimer’s disease (AD) is a disorder characterized by the progressive degeneration of the structure and function of the central nervous system (Plaingam et al., 2017). Globally, individuals with this disorder have steadily increased in numbers, where in 2016, there were 43.8 million cases reported, which is expected to increase to 131.5 million by 2050. In Malaysia, it is estimated that in 2016, there were about 50,000 individuals diagnosed with AD, and this number is projected to increase to 123,000 by 2030 (Feigin et al., 2019). AD is an irreversible disease where it will slowly affect memory and thinking skills. If the disease is not properly managed, the person diagnosed with AD may not be able to carry out daily activities (Nazarko, 2019). In 2021, drug development for all types of AD suffered from a high attrition rate (around 95%), where 117 agents tested in phases I and II and 36 agents tested in phase III were discontinued (Cummings et al., 2021). In 2020, clinical trials for early-to-mild and mild-to-moderate phases of AD involving semagacestat, bapineuzumab, and solanezumab were halted due to lack of improvement in cognitive function. While these drugs showed good results in phases I and II such as reducing the CSF biomarker level, little or no significant improvement in cognitive and functional endpoints were observed during phase III. An increase in dose did not display a change in the cognitive and functional endpoints but resulted in several severe toxic effects such as vascular edema in bapineuzumab (Huang et al., 2020). Currently, only six drugs are approved by the Food and Drug Administration (FDA), which are donepezil, rivastigmine, galantamine, memantine, combination capsule of memantine with donepezil, and aducanumab (Cummings et al., 2021). Aducanumab is the first disease-modifying drug that has been approved by the FDA in June 2021. Although aducanumab clears the amyloid plaque, its efficacy in improving cognitive function is still lacking in evidence (Walsh et al., 2021). This highlights the insufficient evidence of the clinical benefit as well as the narrow therapeutic index of potential AD drugs as some of the challenges faced in the development of AD drugs (Mehta et al., 2017).

The neuronal cell death that occurs in AD is due to the molecular and cellular changes in the brain. It is characterized by the presence of extracellular amyloid-β (Aβ) proteins in senile plaques and intracellular deposits of tau (τ) proteins in neurofibrillary tangles (NFTs) that affect memory and cognition (Hampel et al., 2020). Amyloid precursor protein (APP) is an integral membrane protein found in various tissues, such as synapses of neurons. APP acts as a regulator of synaptic formation and repair, anterograde neuronal transport, and iron export. In healthy neurons, APP is cleaved by an enzyme known as α-secretase through the non-amyloidogenic pathway and produces amyloid precursor protein alpha-secretase (APPsα) ectodomain and membrane-bound carboxyl-terminal fragment (CTF) known as C83. Then, C83 is cleaved by γ-secretase to form p3 and APP intracellular domain (AICD) (Chen et al., 2017). The activity of α-secretase is associated with proteases such as tumor necrosis factor-alpha converting enzyme (TACE), disintegrin, and metalloproteinase domain-containing protein 9 (ADAM9) and ADAM10 (Chen et al., 2017).

However, in AD, β-secretase enzyme (BACE1) and γ-secretase cleave APP to form amyloid beta (Aβ), an insoluble peptide through the amyloidogenic pathway. APP is first cleaved by BACE1 to produce two protein fragments, which are APPsβ (secreted ectodomain) and C99 (membrane-bound fragment) (Ashrafian et al., 2020). C99 will then be cleaved by γ-secretase, which is composed of proteins such as anterior pharynx defective 1 (APH1), presenilin enhancer 2 (PEN2), nicastrin and presenilin (PS1 or PS2), and forms AICD and *C*-terminus of Aβ peptides. The insoluble Aβ peptide, which is made up of 42 amino acids, can aggregate and form amyloid plaques in the brain, a pathological hallmark of AD (Ashrafian et al., 2020). Hence, inhibiting BACE1 could interfere in the formation of amyloid plaque since it is the initial and rate-limiting step of Aβ production. Several inhibitors against BACE1 have been developed over the past few years (Abeysinghe et al., 2020; Hampel et al., 2021; Zhumanova et al., 2021); however, most of them have failed during preclinical trials. BACE1 has a large catalytic pocket, and hence the inhibitors need to be large enough to interact with key amino acid residues of the BACE1 active site (Coimbra et al., 2018). However, large-sized inhibitors may not be able to cross the blood–brain barrier (BBB), as only low molecular weight and lipophilic compounds are able to cross the BBB, unless aided by transporters (Pardridge et al., 2005; Banks, 2009).

BACE1 is structurally homologous to other aspartic proteases of the pepsin family such as BACE2 and cathepsin D (CTSD) where the inhibition of these enzymes can increase the production of Aβ and protein deposits in several organs. Hernández et al. (2016) performed alignment studies, molecular dynamics simulations, and docking studies on these three aspartic proteases to study their differences. Based on the result, selective BACE1 inhibition can be achieved through strong electrostatic interactions with Asp32 and Asp228 of the catalytic site, in addition to a large number of hydrogen bonds, π–π interaction, and van der Waals interaction.

BACE1 is also limited by its highly flexible catalytic site. It has been observed that the presence or absence of an inhibitor within the active site influences the flap’s conformation in BACE1. A better understanding of the binding of substrates and inhibitors to BACE1 is required before developing an inhibitor (Xu et al., 2012). Chakraborty et al. (2011) studied the dynamic transition of BACE1 employing normal mode analysis (NMA) using a simplified elastic network model (ENM). The dynamic transition of BACE1 from an open to a closed conformation can be observed from this study using the combined approach of cavity and volume calculation obtained from the dynamics of BACE1 encoded by normal mode. In the open conformation, a large catalytic cavity allowed the binding of a wide range of substrates with the help of the 10s and F loops. Both loops are part of the cavity, and the F loop forms the C-terminal lining of the cavity. Meanwhile, in the closed conformation, the F loop detaches from the cavity, and the 10s loop moves upward and lines the cavity, which causes the cavity to squeeze and tightly hold the substrate. The effect of myricetin on the BACE1 enzyme was also studied to explain the pharmacophoric feature of BACE1 inhibitors using molecular docking. The docking results showed that myricetin forms a strong interaction with BACE1 in the closed conformation compared to that in the open conformation. In the closed conformation of BACE1, myricetin formed interactions with the enzyme through hydrogen bonding by binding to the catalytic aspartic acid residues (Asp228) and forming van der Waals interactions with Asp32 and Ser35. Myricetin also binds with other residues such as Pro70, Val69, Tyr71, and Thr72 through hydrogen bonds.

In addition, BACE1 inhibitors also need to overcome potential drug–drug interactions from activity against CYP450 enzymes and potential hERG channel inhibition. Inhibition of the hERG channel could cause QT interval prolongation that may lead to ventricular arrhythmias known as Torsades de Pointes (TdP) (Coimbra et al., 2018). Malone and Hancox (2020) evaluated evidence from the scientific literature to find potential association between AD drugs (donepezil, galantamine, and rivastigmine) and the risk of QT interval prolongation and Torsades de Pointes (TdP). From the study, one of the AD drugs, which is donepezil, showed a risk of producing QT interval prolongation and Torsades de Pointes (TdP).

Natural product compounds may be potential candidates for BACE1 inhibitors. Several natural product compounds have been shown to bind simultaneously to the catalytic amino acid residues of BACE1, which are Asp228 and Asp32. Wagle et al. (2019) performed molecular docking between BACE1 (pdb id: 2wjo) and isolated compounds from *Cirsium japonicum* var. maackii, namely luteolin, luteolin 5-O-β-D-glucopyranoside, and luteolin 7-O-β-D-glucopyranoside. The result showed that luteolin 5-O-β-D-glucopyranoside and luteolin 7-O-β-D-glucopyranoside interacted with both Asp228 and Asp32 residues *via* hydrogen bonds. Additionally, natural product compounds have low activity against the hERG channel due to the presence of aromatic rings (Kratz et al., 2017; Babiaka et al., 2020). Moreover, natural product compounds have the ability to cross the blood–brain barrier (BBB) as they have physicochemical properties that are favorable for BBB permeation (Carecho et al., 2020). Low-molecular-weight polyphenol molecules can cross the BBB through passive permeation and carrier-mediated transport. Flavonoids are able to cross the BBB through transporters such as ATP-binding cassette (ABC) transporters, organic anion transporters (OATs), and organic anion transporting polypeptides (OATPs) (Lu et al., 2014; Stieger et al., 2017; Williamson et al., 2018).

**FIGURE 1:**
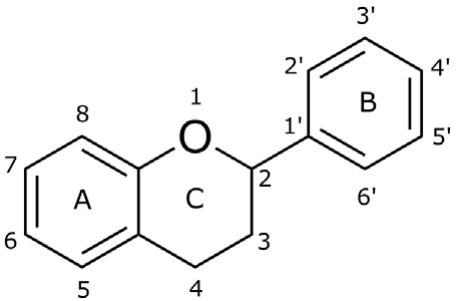
Main structure of flavonoid, which contains two aromatic rings (A and B) and linked *via* a heterocyclic ring (ring C).

One of the essential classes of natural product compounds is flavonoids. It can be found in several parts of a plant and is a secondary metabolite. The main structure of flavonoids contains two aromatic rings (ring A and ring B) connected by three carbon atoms, which form an oxygenated heterocycle (ring C) (Figure 1). There are about eight subclasses of flavonoids, which are flavones, flavonols, flavanols, isoflavonoids, chalcones, anthocyanins, flavanones, and neoflavanoids. The subclasses are categorized based on the basic flavan ring system, alkylation, glycosylation, and hydroxylation (Vauzour et al., 2008; Panche et al., 2016). The presence of chromone structure in the backbone of many flavonoids is an important feature in several anti-HIV, anti-inflammatory, antibacterial, and anticancer drugs as well as those used in neurodegenerative diseases, inflammatory diseases, and diabetes (Reis et al., 2017).

Several flavonoids from various plants such as mycertin, kaempferol, morin, apigenin, luteolin, and polymethoxyflavones have been studied for their inhibitory activity against BACE1. A study from Youn et al. (2016a) showed that 5,7-dimethoxyflavone (DMF), 5,7,4′-trimethoxyflavone (TMF), and 3,5,7,3′,4′-pentamethoxyflavone (PMF) exhibited strong BACE1 inhibitory activities with no suppression of other enzyme activities such as α-secretase and other serine proteases. This indicates that these three flavonoid compounds are relatively specific and selective toward the BACE1 enzyme. Moreover, the study by Zhumanova et al. (2021) also reported that O-methylated quercetins, a flavonoid isolated from the aerial part of the endemic *Caragana balchaschensis* (Kom.) Pojark, were significantly effective in inhibiting BACE1 with IC_50_ values ranging from 1.2 to 6.5 μM. Additionally, flavonoids such as kaempferol and quercetin have diverse bioactivities and are known to exert neuroprotective effects and inhibit BACE1 activity. A number of flavonoids have also been investigated for anti-acetylcholinesterase (AChE) and butyrylcholinesterase (BChE) activities. In a study, macluraxanthone was found to be the most potent and specific inhibitor of both AChE and BChE, with IC_50_ values of 8.47 and 29.8 μM, respectively (Khan et al., 2009).

Despite their favorable structure and properties, to date, no flavonoids have advanced further in clinical trials as BACE1 inhibitors. Hence, the aim of this study was to analyze the data published between 2010 and 2022 regarding flavonoids and their activity against the BACE1 enzyme. Specifically, the two-dimensional (2D) structural patterns as well as binding patterns of the flavonoids are analyzed, with the aim of discovering the functional relationship between structure and activity. Understanding the structure–activity relationship between flavonoids and BACE1 is important in designing highly potent and specific BACE1 inhibitors.

## Materials and Methods

In the data collection that is similar to that of a systematic review process, two authors (Nur Intan Saidaah; NS, and (Zafirah Liyana Abdullah; ZA) independently reviewed and evaluated all articles found in the literature search, including titles, abstracts, and keywords based on the inclusion and exclusion criteria. If there was any disagreement between the authors about the articles reviewed, a third author (Fazlin Mohd Fauzi; FF) reviewed the article and made the final decision.

### Keyword Identification

A comprehensive literature search was performed through several databases such as Science Direct, Scopus, and PubMed using search strings (Table 1). The search was limited to articles published from 2010 to 2022, and only research and journal articles were selected as the document type. In this study, 389 articles were retrieved in the first round of electronic literature searches. After removing review papers, conference proceedings, letters, meta-analyses, abstracts, emails, and unrelated topics (*n* = 299), 90 articles were identified for screening.

**TABLE 1:** Search string used for each database to search for relevant literatures.

Database search string	
Science Direct	Alzheimer’s disease AND (β-secretase OR bace1) AND (inhibitors OR inhibition) AND flavonoids
Scopus	[TITLE-ABS-KEY (Alzheimer’s AND disease) AND TITLE-ABS-KEY (β-secretase) OR TITLE-ABS-KEY (bace1) AND TITLE-ABS-KEY (inhibitors) OR TITLE-ABS-KEY (inhibition) AND TITLE-ABS-KEY (flavonoids)] AND DOCTYPE (ar) AND PUBYEAR > 2010 AND PUBYEAR < 2020
PubMed	((((Alzheimer’s disease [MeSH Terms]) AND (β-secretase)) OR (bace1) AND ((journal article [Filter]) AND (2010:2020[pdat]))) AND (inhibitors)) AND (flavonoids)

### Screening

In the screening stage, 90 articles were screened based on their titles and abstracts. The articles were screened based on the following criteria: 1) studies that did not include the BACE1 enzyme inhibition assay and 2) studies that did not involve flavonoids as compounds against BACE1. Duplicate articles were also removed. A total of 74 articles were excluded based on these criteria.

### Eligibility

A total of 16 articles were evaluated for their eligibility. All articles were examined based on inclusion and exclusion criteria (Table 2). Four studies were excluded because the studies either involved plant extracts, only *in silico* experiments were conducted without *in vitro* validation, no full text was available, and/or IC_50_ values that were not available in M (Table 2). Finally, 12 studies with 64 flavonoids were selected after 2 duplicates were removed.

**TABLE 2:** Inclusion and exclusion criteria used in the screening phase of the literature search.

Inclusion criteria
Evaluation of the BACE1 enzyme inhibition assay
Studies involve flavonoid compounds against the BACE1 enzyme
Design of the study includes *in vitro* evaluation. If *in silico* studies were present, it must be supplemented with *in vitro* validation
Full text available
Exclusion criteria
Review articles, conference proceedings, letters, meta-analyses, abstracts, e-mails, and studies on humans
Studies involving plant extracts against the BACE1 activity
*In silico* studies without *in vitro* validation

### Data Extraction

Information of all 64 flavonoids from 12 studies were extracted, which include authors, year of publication, the structure of flavonoids, and BACE1 inhibitory activities. A chemical annotation of the flavonoids such as SMILES was identified and tabulated in an Excel spreadsheet, which was then converted into a CSV format for further analysis.

### Molecular Properties and Clustering

The molecular properties of the flavonoids were calculated based on their structure in DataWarrior (www.openmolecules.org). Next, the flavonoids were clustered based on their chemical structure. All of the extracted information in the CSV file was uploaded into the DataWarrior software. Under the Chemistry tab, the Calculate Properties was selected. Properties for drug-likeness such as cLogP, H-acceptors, H-donors, and total surface area were chosen. Automatic structure–activity relationship (SAR) analysis was performed under the Chemistry tab to determine core fragments and functional groups of flavonoid compounds. In this analysis, SMILES was used to generate the scaffold, with Murcko scaffold selected as the scaffold type. After core fragments and functional groups were determined, a cluster analysis was performed. In the same Chemistry tab, the Cluster Compounds or Reactions was selected. Clustering starts with the construction of a complete similarity matrix of all flavonoids that was calculated based on the default descriptor, *FragFp*, which is similar to MDL keys and consists of 512 predefined structural fragments*.* The flavonoids with the highest similarity were included in the first cluster. Then, the similarity matrix is updated by removing all similarity values related to the first two flavonoids. This is replaced by a new set of similarities between the cluster center and other compounds. The new values were calculated as mean of the two original similarity values. The process continues by merging the most similar flavonoid and terminated when the highest similarity falls below 0.8.

### Molecular Docking

Representative compounds from each cluster were subjected to molecular docking against BACE1. The crystal structure of BACE1 (pdb id: 2wjo, 2.50 Å) was obtained from the Research Collaboratory for Structural Bioinformatics (RCSB) Protein Data Bank (PDB). Using Discovery Studio Visualizer Software, heteroatoms and water were removed, and polar hydrogen was added to the BACE1 structure. BACE1 was loaded onto the PyRx software and set as a macromolecule. The three-dimensional (3D) structures of each representative compound and the reference ligand (2,2,4-trihydroxychalcone; CID: 5811533) were obtained from the PubChem Compound Database (NCBI). These files were uploaded to the Open Babel tab in PyRx software, and energy minimization was selected for all the ligands. The force field used in the Open Babel software package is by default the universal force field. The docking of the BACE1 and flavonoid compounds was simulated using PyRx Software (AutoDock Vina) to determine the binding energy for each flavonoid with BACE1. The Lamarckian genetic algorithm (LGA) method was used for all calculations between the protein and ligand. The docking site on the protein target was defined by establishing a grid box with the dimensions of X: 61.0616, Y: 51.4261, and Z: 59.9516 Å, with a center of X: 16.0076, Y: 39.7409, and Z: 40.5572 Å. The visualization of BACE1 with flavonoid compounds was visualized using Discovery Studio Visualizer (Trott and Olson, 2010; Dallakyan and Olson, 2015).

## Results

### General Findings

**FIGURE 2:**
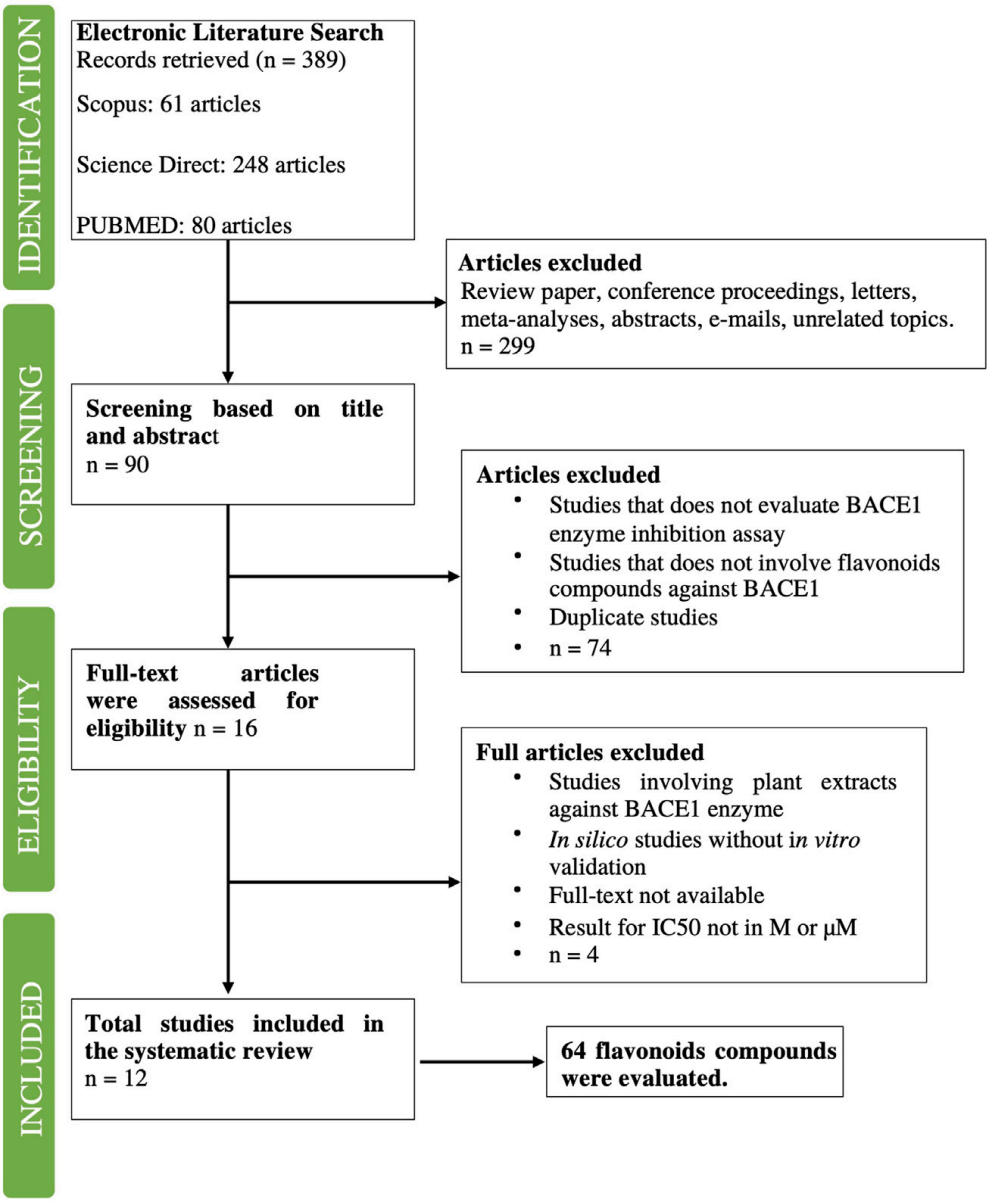
Flowchart of the literature search conducted and number of articles filtered at each stage. The process is similar to that of a systematic review.

From the 12 articles that were filtered, 66 flavonoids were collected and included information such as chemical structure, molecular properties, and IC_50_ values. Two duplicates were found and removed, which were **naringenin** and **baicalein**. In total, 64 compounds were filtered (Figure 2). For each of the 64 flavonoids, their core fragments were identified, which include flavone, flavanone, chalcone, rotenoid, and isoflavone. For one compound, its core fragment could not be identified, which was **neocyclomorusin**.

### Clustering Analysis Based on Core Fragment and Functional Groups of Flavonoids

**FIGURE 3:**
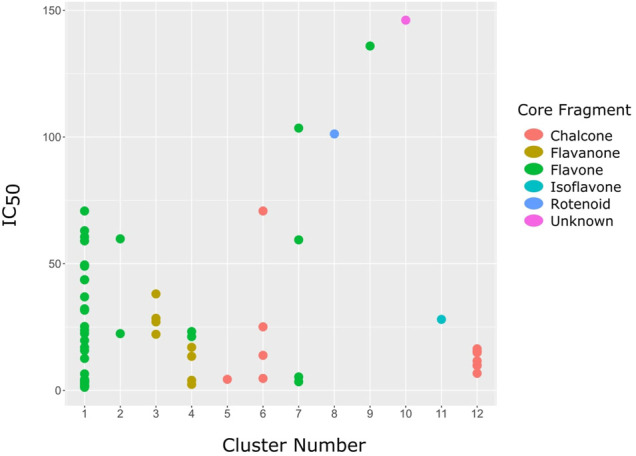
The graph showing the distribution of IC_50_ values for compounds in each cluster. The graph shows that each cluster contain one distinct core fragment, with the exception of Cluster 4, which contains both Flavone and Flavanone. Flavanone and Chalcone showed the obvious correlation between structure and bioactivity. Flavanones in Cluster 4 contain sugar moieties but is absent in Flavanones in Cluster 3, and the IC_50_ values of both clusters do not overlap.

Using DataWarrior, the 64 flavonoids were clustered based on the similarity of their structures, which resulted in 12 clusters. A full detail of each compound and their cluster is listed in Table 3, and a graph showing the distribution of the IC_50_ for each compound in the cluster is shown in Figure 3. Each cluster contains only compounds with one distinct core fragment, with the exception of cluster 4 where there are compounds with either a flavanone or flavone core fragment, as well as the presence of at least one sugar moiety. Flavonoids from these 12 studies exhibited IC_50_ values ranging from 1.2 to 147 μM against the BACE1 enzyme.

**TABLE 3:** Information of the 64 flavonoid compounds that were filtered and classified according to their cluster.

Structure	Name	(IC_50_; μM)	Core fragment	MW	cLogP	HBA	HBD	TSA	Reference
CLUSTER 1									
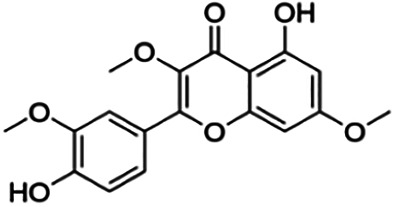	3,7,3′-Tri-O-methylquercetin (Pachypodol)	1.20	Flavone	344.32	2.47	7	2	249.67	Zhumanova et al. (2021)
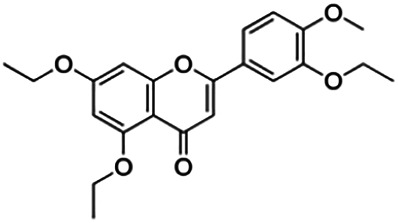	3,5,7-Triethoxy-4 -methoxyflavone (D6)	1.58	Flavone	384.43	4.31	6	0	302.30	Tran et al. (2020)
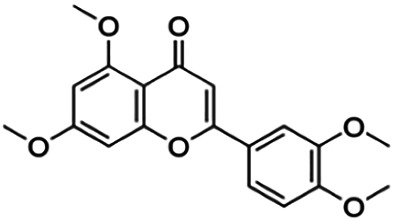	3′,4′,5,7-Tetramethoxyflavone (D5)	1.66	Flavone	342.35	3.09	6	0	261.02	Tran et al.(2020)
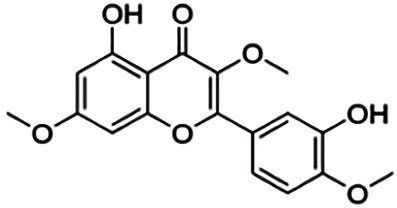	3,7,4′-Tri-O-methylquercetin (Ayanin)	1.80	Flavone	344.32	2.47	7	2	249.67	Zhumanova et al. (2021)
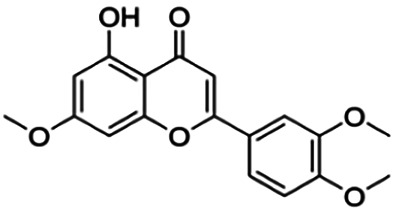	5-Hydroxy-3′,4′,7-trimethoxyflavone (D2)	2.14	Flavone	328.32	2.82	6	1	245.11	Tran et al. (2020)
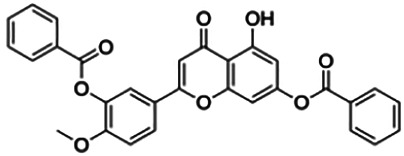	3′,7-Dibenzoyloxy-5-hydroxy-4′-methoxyflavone (D7)	2.86	Flavone	508.48	5.82	8	1	375.61	Tran et al. (2020)
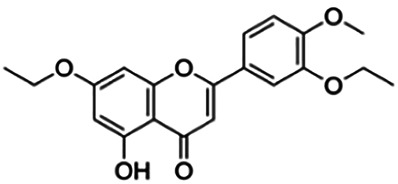	**3′,7-Diethoxy-5-hydroxy-4′-methoxyflavone (D3)**	3.16	Flavone	356.37	3.63	6	1	272.63	Tran et al. (2020)
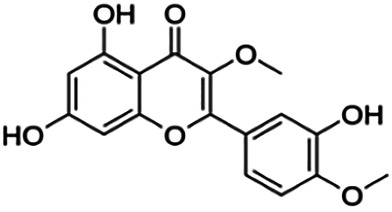	3,4′ -Di-O-methylquercetin	3.50	Flavone	330.29	2.19	7	3	233.76	Zhumanova et al. (2021)
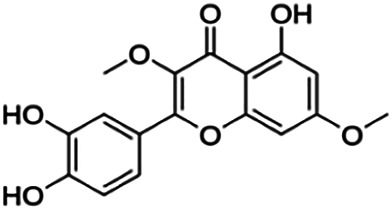	3,7-Di-O-methylquercetin	3.80	Flavone	330.29	2.19	7	3	233.76	Zhumanova et al. (2021)
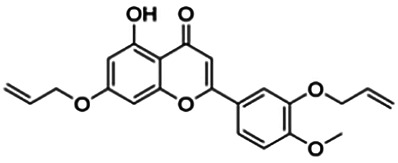	3′,7-Diallyloxy-5-hydroxy-4′-methoxyflavone (D4)	3.60	Flavone	380.40	4.17	6	1	297.19	Tran et al. (2020)
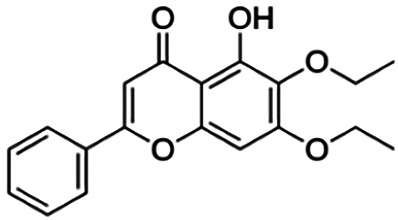	6,7-Diethoxy-5-hydroxyflavone (B3)	3.98	Flavone	326.35	3.70	5	1	250.37	Tran et al. (2020)
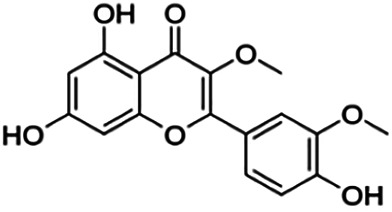	3,3′ -Di-O-methylquercetin	4.30	Flavone	330.29	2.19	7	3	233.76	Zhumanova et al. (2021)
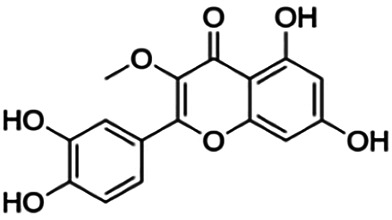	3-O-methylquercetin	6.50	Flavone	316.26	1.92	7	4	217.85	Zhumanova et al. (2021)
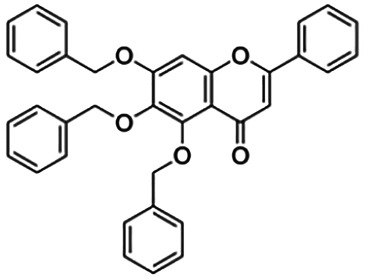	5,6,7-Tribenzyloxyflavone (B6)	12.59	Flavone	540.61	7.42	5	0	422.54	Tran et al. (2020)
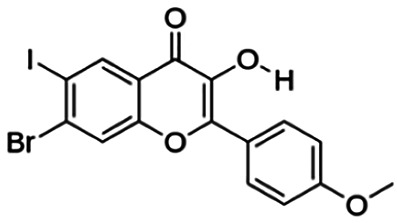	7-Bromo-3-hydroxy-6-iodo-2-(4-methoxyphenyl)-4H-chromen-4-one (3l)	15.74	Flavone	473.06	3.97	4	1	243.71	Mphahlele et al. (2018)
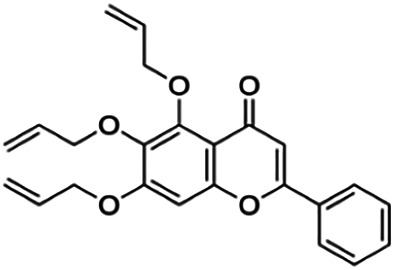	5,6,7-Triallyloxyflavone (B5)	16.98	Flavone	390.43	5.19	5	0	316.88	Tran et al. (2020)
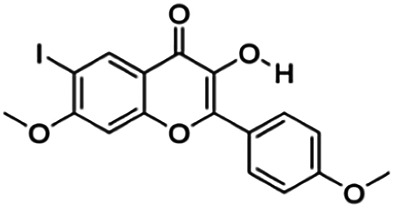	2-(4-Chlorophenyl)-7-fluoro-3-hydroxy-6-iodo-4H-chromen-4-one (3c)	19.69	Flavone	416.57	4.02	3	1	224.59	Mphahlele et al. (2018)
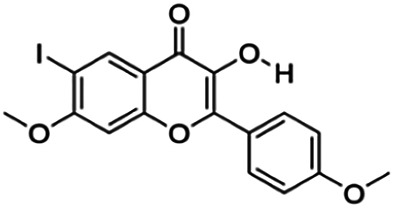	3-Hydroxy-6-iodo-7-methoxy-2-(4-methoxyphenyl)-4H-chromen-4-one (3p)	22.44	Flavone	424.19	3.17	5	1	247.34	Mphahlele et al. (2018)
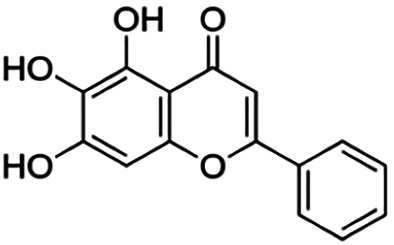	Baicalein	23.71	Flavone	270.24	2.34	5	3	191.03	Han et al. (2019)
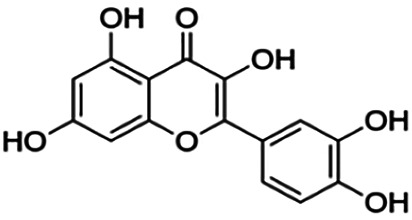	Quercetin	25.20	Flavone	302.24	1.49	7	5	201.94	Zhumanova et al. (2021)
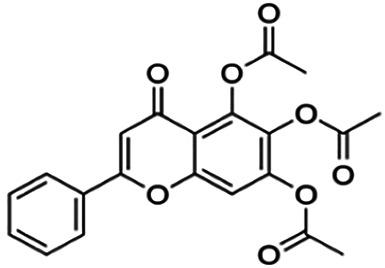	5,6,7-Triacetoxyflavone (B7)	31.62	Flavone	396.35	3.33	8	0	292.01	Tran et al. (2020)
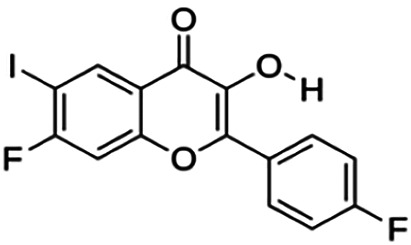	7-Fluoro-2-(4-fluorophenyl)-3-hydroxy-6-iodo-4H-chromen-4-one (3b)	32.18	Flavone	400.11	3.51	3	1	215.52	Mphahlele et al. (2018)
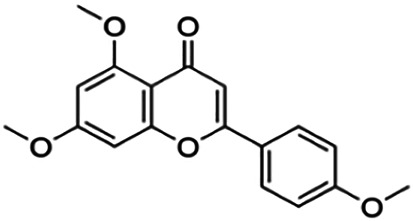	5,7,4′-Trimethoxyflavone	36.90	Flavone	282.29	3.16	5	0	238.76	Youn et al. (2016a)
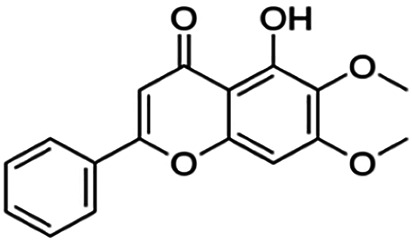	5-Hydroxy-6,7-dimethoxyflavone (B2)	43.65	Flavone	298.29	2.89	5	1	222.85	Tran et al. (2020)
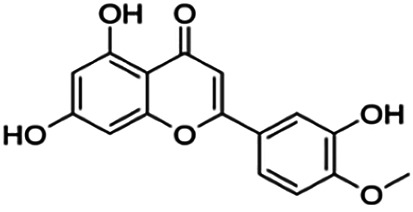	Diomestin (D1)	43.65	Flavone	462.41	2.27	6	3	213.29	Tran et al. (2020)
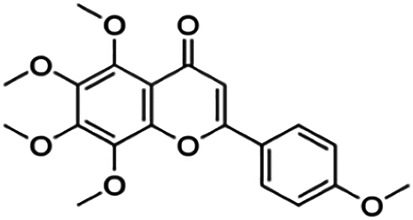	Tangeretin	49.00	Flavone	372.37	3.02	7	0	283.28	Youn et al. (2017)
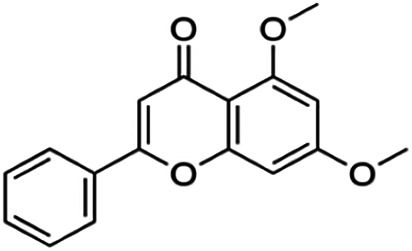	5,7-Methoxyflavone	49.50	Flavone	312.32	3.23	4	0	216.50	Youn et al. (2016a)
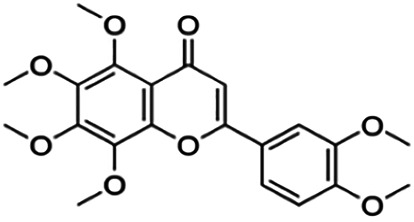	Nobiletin	59.00	Flavone	402.40	2.95	8	0	305.54	Youn et al. (2017)
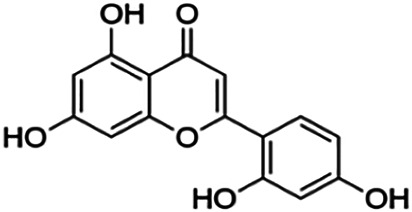	Norartocarpetin	60.60	Flavone	286.24	1.99	6	4	197.38	Cho et al. (2011)
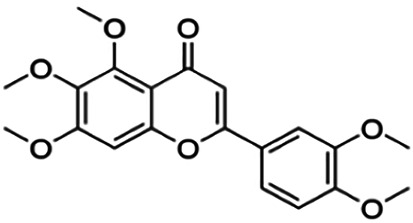	Sinensetin	63.00	Flavone	372.37	3.02	7	0	283.28	Youn et al. (2017)
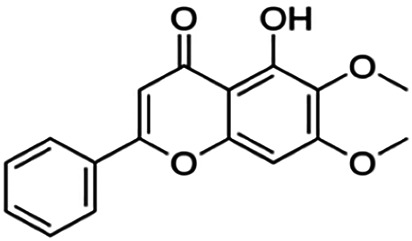	6,7-Diallyloxy-5-hydroxyflavone (B4)	70.79	Flavone	298.29	2.89	5	1	222.85	Tran et al. (2020)
CLUSTER 2									
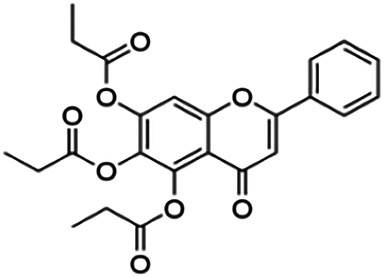	5,6,7-Tripropionoxyflavone (B8)	22.39	Flavone	438.43	4.70	8	0	333.29	Tran et al. (2020)
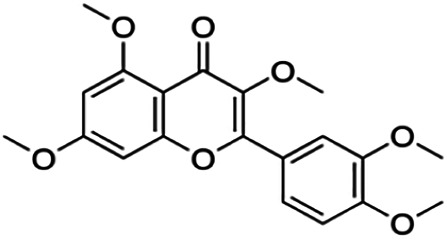	**3,5,7,3′,4′-Pentamethoxyflavone**	59.80	Flavone	372.37	3.02	7	0	281.49	Youn et al. (2016a)
CLUSTER 3									
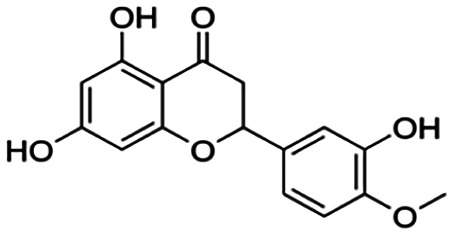	Hesperetin	22.13	Flavanone	302.28	2.09	6	3	214.84	Lee et al. (2018)
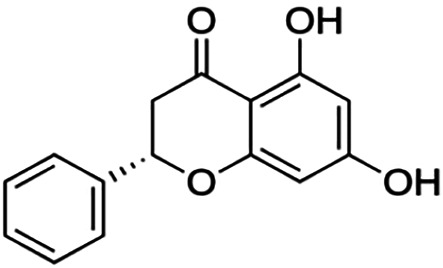	Pinocembrin	27.01	Flavanone	256.26	2.50	4	2	186.23	Youn and Jun (2019)
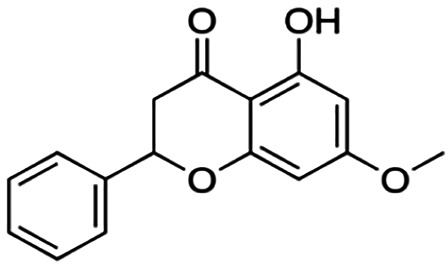	Pinostrobin	28.44	Flavanone	270.28	2.78	4	1	202.14	Youn and Jun (2019)
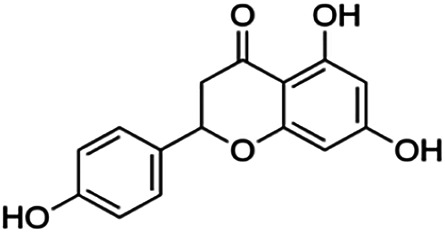	**Naringenin**	38.06	Flavanone	272.26	2.16	5	3	192.58	Ali et al. (2019)
CLUSTER 4									
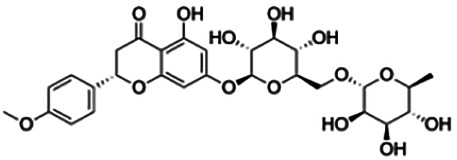	**Didymin**	2.34	Flavanone	594.56	−0.47	14	7	404.26	Ali et al. (2019)
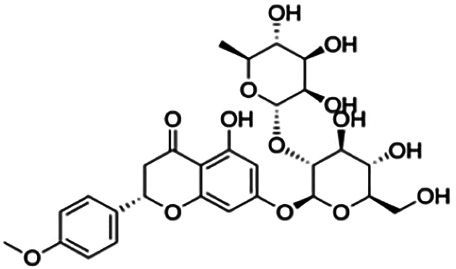	Poncirin	3.96	Flavanone	594.56	−0.47	14	7	404.26	Ali et al. (2019)
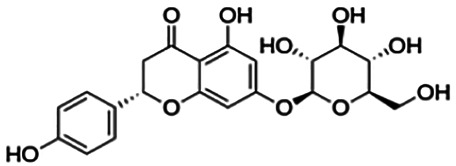	Prunin	13.41	Flavanone	434.40	0.17	10	6	294.39	Ali et al. (2019)
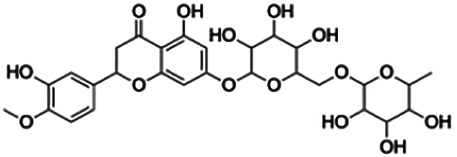	Hesperidin	16.99	Flavanone	610.56	−0.81	15	8	410.61	Lee et al. (2018)
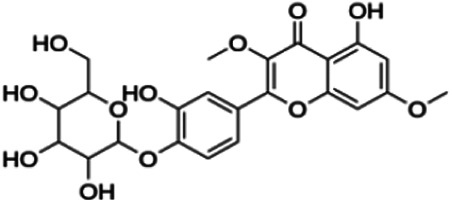	3,7-Di-O-methylquercetin-4′ -O-glucoside	21.20	Flavone	492.43	0.21	12	6	335.57	Zhumanova et al. (2021)
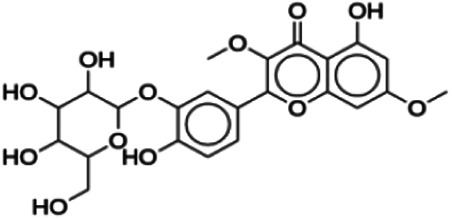	3,7-Di-O-methylquercetin-3′-O-glucoside	23.20	Flavone	492.43	0.21	12	6	335.57	Zhumanova et al. (2021)
CLUSTER 5									
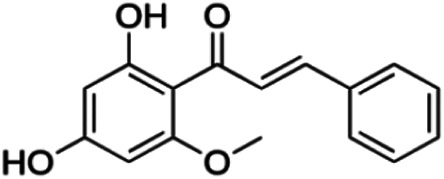	**Cardamonin**	4.35	Chalcone	270.28	2.5424	4	2	212.23	Youn and Jun (2019)
CLUSTER 6									
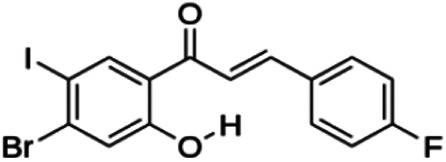	(E)-1-(4-bromo-2-hydroxy-5-iodophenyl)-3-(4-fluorophenyl)propenone (2j)	4.70	Chalcone	447.04	4.22	2	1	234.88	Mphahlele et al. (2018)
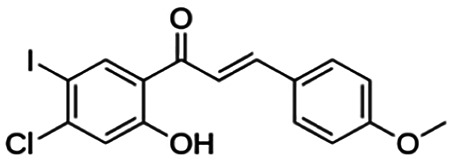	(E)-1-(4-chloro-2-hydroxy-5-iodophenyl)-3-(4-methoxyphenyl)propenone (2h)	13.82	Chalcone	414.62	3.93	3	1	247.58	Mphahlele et al. (2018)
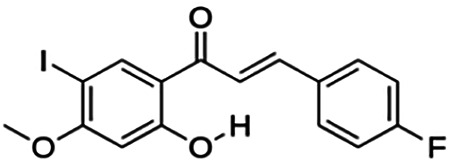	**(E)-3-(4-fluorophenyl)-1-(2-hydroxy-5-iodo-4-methoxyphenyl)prop-2-en-1-one (2n)**	25.07	Chalcone	398.17	3.43	3	1	238.51	Mphahlele et al. (2018)
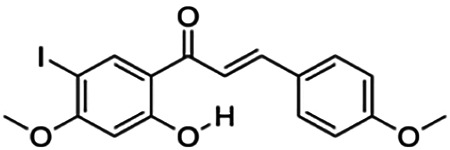	(E)-1-(2-hydroxy-5-iodo-4-methoxyphenyl)-3-(4-methoxyphenyl)prop-2-en-1-one (2p)	70.79	Chalcone	410.20	3.26	4	1	254.42	Mphahlele et al. (2018)
CLUSTER 7									
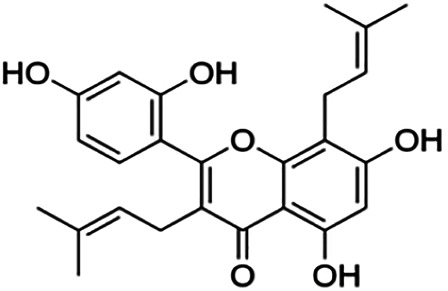	Kuwanon C	3.40	Flavone	422.48	6.17	6	4	321.57	Cho et al. (2011)
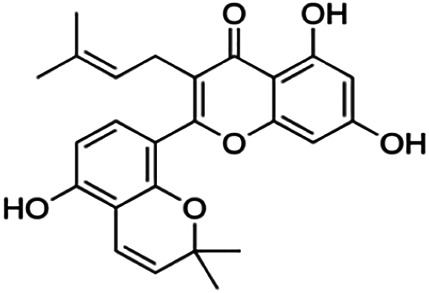	Kuwanon A	5.30	Flavone	420.46	5.52	6	3	310.16	Cho et al. (2011)
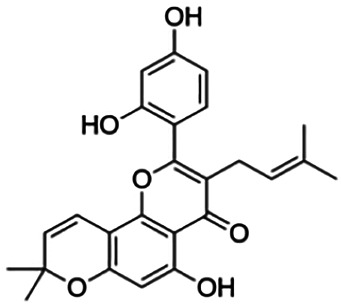	**Morusin**	59.40	Flavone	420.46	5.52	6	3	310.16	Cho et al. (2011)
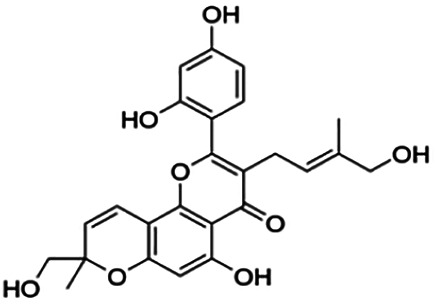	Mormin	103.50	Flavone	452.46	3.67	8	5	325.86	Cho et al. (2011)
CUSTER 8									
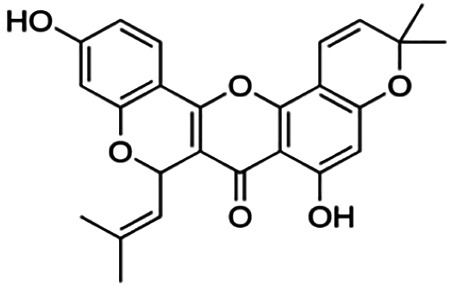	**Cyclomorusin**	101.20	Rotenoid	418.44	5.24	6	2	299.05	Cho et al. (2011)
CLUSTER 9									
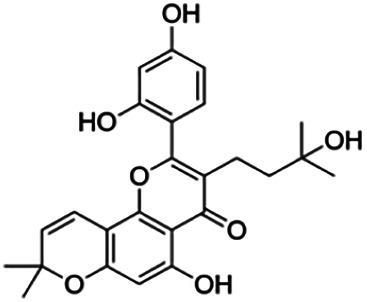	**Morusinol**	135.90	Flavone	438.47	4.52	7	4	315.97	Cho et al. (2011)
CLUSTER 10									
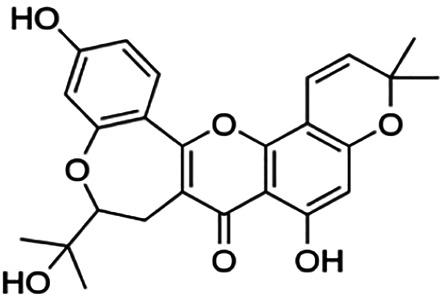	**Neocyclomorusin**	146.10	Unknown	436.46	4.13	7	3	304.86	Cho et al. (2011)
CLUSTER 11									
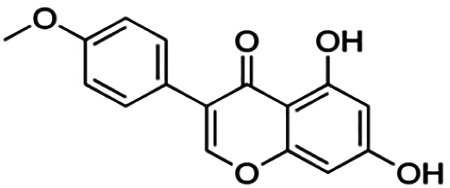	**Biochanin A**	28	Isoflavone	284.266	1.9029	5	2	206.94	Youn et al. (2016b)
CLUSTER 12									
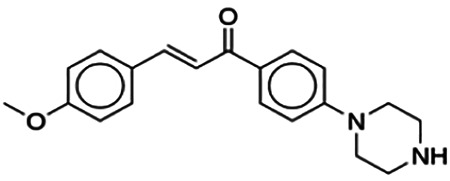	(2E)-3-(4-Methoxyphenyl)-1-[4-(1-piperazinyl)phenyl]-2-propen-1-one (PC3)	6.72	Chalcone	322.41	2.76	4	1	262.83	Mathew et al. (2021)
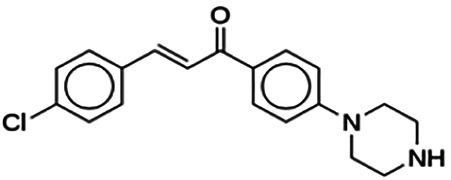	(2E)-3-(4-Chlorophenyl)-1-[4-(1-piperazinyl)phenyl]-2-propen-1-one (PC8)	9.76	Chalcone	324.85	4.49	2	1	260.99	Mathew et al. (2021)
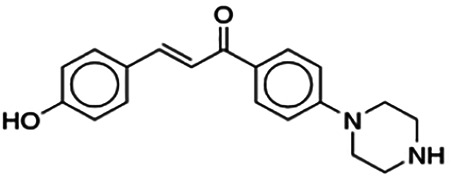	(2E)-3-(4-Hydroxyphenyl)-1-[4-(1-piperazinyl)phenyl]-2-propen-1-one (PC2)	9.86	Chalcone	308.38	4.83	4	2	246.92	Mathew et al. (2021)
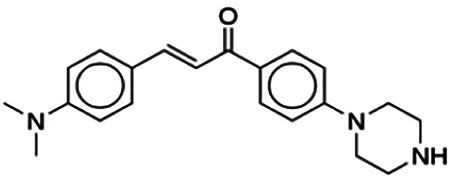	(2E)-3-(4-Dimethylaminophenyl)-1-[4-(1-piperazinyl)phenyl]-2-propen-1-one (PC5)	11.60	Chalcone	335.45	2.73	4	1	275.39	Mathew et al. (2021)
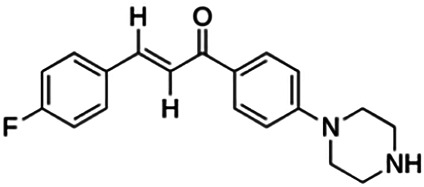	(2E)-3-(4-Fluorophenyl)-1-[4-(1-piperazinyl)phenyl]-2-propen-1-one (PC10)	14.90	Chalcone	308.40	3.98	2	1	251.92	Mathew et al. (2021)
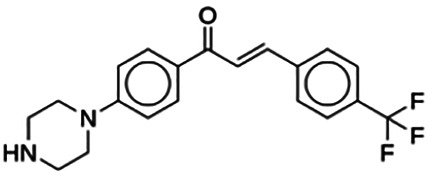	(2E)-3-(4-Trifluorophenyl)-1-[4-(1-piperazinyl)phenyl]-2-propen-1-one (PC11)	15.30	Chalcone	360.38	3.68	3	1	270.03	Mathew et al. (2021)
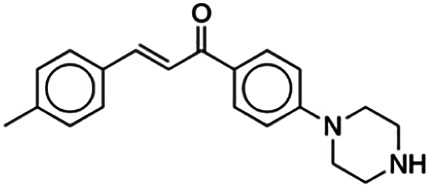	**(2E)-3-(4-Methylphenyl)-1-[4-(1-piperazinyl)phenyl]-2-propen-1-one (PC4)**	15.50	Chalcone	306.41	3.17	3	1	252.83	Mathew et al. (2021)
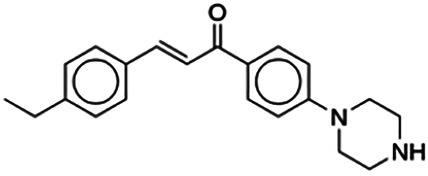	(2E)-3-(4-Ethylphenyl)-1-[4-(1-piperazinyl)phenyl]-2-propen-1-one (PC6)	16.40	Chalcone	320.44	3.59	3	1	266.59	Mathew et al. (2021)

Information includes chemical structure, IC_50_ value, molecular weight, cLogP, number of hydrogen bond donors and acceptors, and total surface area. Representative compounds for each cluster are highlighted in bold. Abbreviation: MW, molecular weight; cLog, calculated Log P; HBA, hydrogen bond acceptor; HBD, hydrogen bond donor; TSA, total surface area

From Figure 3, it can be seen that clusters 1, 2, 7, and 9 contain compounds with the flavone core exclusively with the highest number of compounds in cluster 1 (31 compounds). Additionally, cluster 4 also contains two compounds with the flavone core. The standard structure of flavone consists of a double bond between C2 and C3, with a ketone at C4. The flavones showed cLogP values of 0.21–7.5, a total surface area of 191–422 m^2^/g, and a molecular weight of 270–541 g/mol. Flavones in clusters 1, 2, 7, and 9 contain around 0–5 hydrogen bond donors and 3–8 hydrogen acceptors, but flavones in cluster 4 contain 6 hydrogen bond donors and 12 hydrogen bond acceptors. The higher hydrogen bond acceptors and donors of compounds in cluster 4 may be attributed to the presence of a sugar moiety. The IC_50_ values of compounds with the flavone core fragment were below 100 μM except for mormin in cluster 7 (103.5 μM) and morusinol in cluster 9 (135.9 μM). Compound **3,7,3′-tri-O-methylquercetin** from cluster 1 showed the lowest IC_50_ value, which was 1.20 μM. **3,7,3′-tri-O-methylquercetin** consists of one hydroxyl group at C5 and one methoxy group at C7 (ring A). It also has a hydroxyl group at C4’ and a methoxy group at C5′ (ring B). In addition, the presence of a methoxy group at C3 (ring C) may contribute to the inhibition of BACE1. It can be seen from Figure 3 that compounds with the flavone core fragment do not have an obvious correlation between the 2D structure and bioactivity, where the clusters show distinct structures but the ranges of IC_50_ values overlap between the clusters.

Cluster 4 is the only cluster that includes compounds with different core fragments where it contains four flavanones and two flavones. Unlike flavone, flavanone compounds have no double bond between C2 and C3, but all compounds in cluster 4 contain at least one sugar moiety. The IC_50_ of compounds in cluster 4 ranges from 2.34 to 23.2 μM where **didymin** in Cluster 4 showed the most potent BACE1 inhibition with an IC_50_ of 2.34 μM, which may be due to the presence of disaccharides at ring A. Although **poncirin** also has disaccharides at ring A, the different position of the glycosidic linkage may explain the IC_50_ value of **poncirin** being slightly higher than that of **didymin**. **Prunin**, which only has one sugar moiety, showed a lower IC_50_ value than **hesperidin**, which has two sugar moieties, similar to **didymin**. The substitution of the hydroxide at ring B at different positions may explain the difference in the IC_50_ value observed. Additionally, the two flavones in the cluster, **3,7-di-O-methylquercetin-4′ -O-glucoside** and **3,7-di-O-methylquercetin-3′-O-glucoside**, had the highest IC_50_ values of 21.2 and 23.2 μM, respectively. Compounds with the flavanone core also can be found in cluster 3 but without the presence of a sugar moiety. The physicochemical properties of flavanone core fragments between clusters 3 and 4 are comparatively different. In cluster 3, the range of cLogP was between 2 and 2.8 (vs. −0.18–0.17) with a total surface area of 186–215 m^2^/g (vs. 294–411 m^2^/g) and a molecular weight ranging from 256 to 303 g/mol (vs. 434–611 g/mol). In cluster 3, IC_50_ values ranged from 22 to 39 μM. When comparing flavanones between clusters 3 and 4, it showed the most obvious relationship between 2D structure and bioactivity where flavanones with sugar moieties produce lower IC_50_ values.

Thirteen flavonoids with the chalcone core fragment were retrieved from the literature screening. Chalcone structures contain an α, β-unsaturated ketone with two aromatic rings (rings A and B). These chalcones can be further categorized as piperazine- and non-piperazine-substituted chalcones. The piperazine-substituted chalcones (8 compounds) were all grouped in cluster 12, while the non-piperazine-substituted chalcones were grouped in clusters 5 (1 compound) and 6 (4 compounds). The SAR of the chalcones was not obvious; however, this relationship is more obvious among the non-piperazine-substituted chalcones. **Cardamonin** (cluster 5) showed the highest inhibitory activity among all chalcones, with an IC_50_ of 4.35 μM. In contrast, compounds in cluster 6 showed IC_50_ above 4.7 μM with compounds **2j** ((E)-1-(4-bromo-2-hydroxy-5-iodophenyl)-3-(4-fluorophenyl)propanone), **2h** ((E)-1-(4-chloro-2-hydroxy-5-iodophenyl)-3-(4-methoxyphenyl)propanone), **2n** ((E)-3-(4-fluorophenyl)-1-(2-hydroxy-5-iodo-4-methoxyphenyl)prop-2-en-1-one), and **2p** ((E)-1-(2-hydroxy-5-iodo-4-methoxyphenyl)-3-(4-methoxyphenyl)prop-2-en-1-one) showing IC_50_ values of 4.703, 13.82, 25.07, and 70.79 μM, respectively. **Cardamonin** contains a hydroxyl group at the *ortho* and *para* positions of ring A and no substitution in ring B. For compounds in cluster 6, all compounds are substituted at the *ortho*, *para,* and *meta* positions of ring A and the *para* position of ring B. All compounds in cluster 6 contain iodine at the *meta* position of ring A. Compound **2j** ((E)-1-(4-bromo-2-hydroxy-5-iodophenyl)-3-(4-fluorophenyl)propanone), which has the lowest IC_50_, is substituted with bromine and fluorine at the *para* position of rings A and B, respectively. Substitution with functional groups with decreasing electronegativity at the *para* position of rings A and B seems to result in an increase in IC_50_ values. When the bromine in **2j** ((E)-1-(4-bromo-2-hydroxy-5-iodophenyl)-3-(4-fluorophenyl)propanone) is replaced with chlorine in ring A and fluorine is replaced with methoxy in ring B, this produces compound **2h** ((E)-1-(4-chloro-2-hydroxy-5-iodophenyl)-3-(4-methoxyphenyl)propanone) with a higher IC_50_ value. When methoxy is substituted at the *para* position of ring A, this produces compounds with higher IC_50_ values, as seen in **2n** ((E)-3-(4-fluorophenyl)-1-(2-hydroxy-5-iodo-4-methoxyphenyl)prop-2-en-1-one) and **2p** ((E)-1-(2-hydroxy-5-iodo-4-methoxyphenyl)-3-(4-methoxyphenyl)prop-2-en-1-one). Both compounds differ in the substitution at the *para* position of ring B, where **2n** ((E)-3-(4-fluorophenyl)-1-(2-hydroxy-5-iodo-4-methoxyphenyl)prop-2-en-1-one) is substituted with a fluorine and recorded a lower IC_50_ value than **2p** ((E)-1-(2-hydroxy-5-iodo-4-methoxyphenyl)-3-(4-methoxyphenyl)prop-2-en-1-one), which is substituted with a methoxy group. This suggests that the potency of non-piperazine-substituted chalcone decreases with the substitution of functional groups with decreasing electronegativity. Additionally, no substitution at ring B seems to correlate to higher activity, and similar to ring A, substitution at the *para* position with functional groups with decreasing electronegativity results in an increase in the IC_50_ value. In terms of the pharmacokinetic profile, the cLogP values for the non-piperazine-substituted chalcone was in the range of 2.5–4.22, with a total surface area of 212–255 m^2^/g and a molecular weight of 270–448 g/mol. The number of hydrogen bond acceptors in these clusters was around 2–4, while the number of hydrogen bond donors was between 1 and 2. In regards to the piperazine-substituted chalcones, although the piperazine-substituted chalcones only differ by the substitution at the *para* position of ring B, no correlation can be established between the properties of the functional groups, i.e., electronegativity or lipophilicity and IC_50_ values. The lowest IC_50_ value in the piperazine-substituted chalcones was observed in compound **PC3** ((2E)-3-(4-methoxyphenyl)-1-[4-(1-piperazinyl)phenyl]-2-propen-1-one; 6.72 μM), where a methoxy is substituted at the *para* position of ring B. This is followed by **PC8** ((2E)-3-(4-chlorophenyl)-1-[4-(1-piperazinyl)phenyl]-2-propen-1-one; 9.76), PC2 ((2E)-3-(4-hydroxyphenyl)-1-[4-(1-piperazinyl)phenyl]-2-propen-1-one; 9.86 μM), **PC5** ((2E)-3-(4-dimethylaminophenyl)-1-[4-(1-piperazinyl)phenyl]-2-propen-1-one; 11.6 μM), **PC10** ((2E)-3-(4-fluorophenyl)-1-[4-(1-piperazinyl)phenyl]-2-propen-1-one; 14.9 μM), PC11 ((2E)-3-(4-trifluorophenyl)-1-[4-(1-piperazinyl)phenyl]-2-propen-1-one; 15.3 μM), **PC4** ((2E)-3-(4-methylphenyl)-1-[4-(1-piperazinyl)phenyl]-2-propen-1-one; 15.5 μM), and **PC6** ((2E)-3-(4-ethylphenyl)-1-[4-(1-piperazinyl)phenyl]-2-propen-1-one; 16.4 μM). In terms of the pharmacokinetic profile, the cLogP values for the piperazine-substituted chalcone was in the range of 2.7–4.9, with a total surface area of 246–276 m^2^/g and a molecular weight of 306–360 g/mol, which are comparable to the non-piperazine-substituted chalcones.

The remaining three clusters contained only one compound each, where **cyclomorusin**, **neocyclomorusin**, and **biochanin A** were clustered in clusters 8, 10, and 11, respectively, each representing a distinct core fragment. **Neocyclomorusin** from cluster 10 showed the highest IC_50_ value, which was 146.1 μM.

### Molecular Docking of Compound Representative From Each Cluster

**FIGURE 4:**
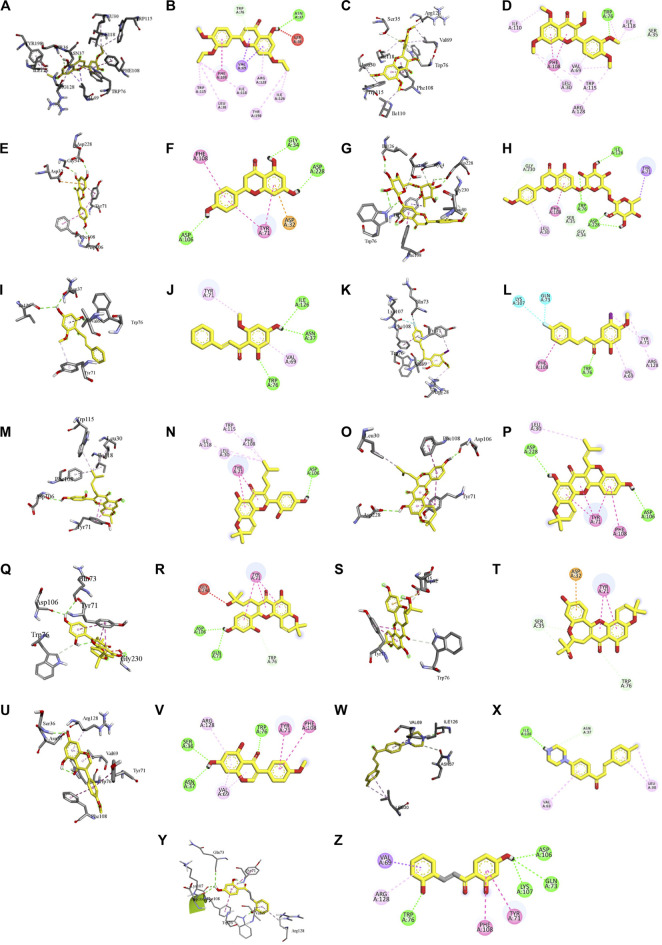
Visualization of the protein–ligand interaction between 11 flavonoids, which represents the 11 clusters against BACE1 from molecular docking. The flavonoids were D3 (3′,7-diethoxy-5-hydroxy-4′-methoxyflavone **(A,B)**, 3,5,7,3′,4′-pentamethoxyflavone **(C,D)**, naringenin **(E,F)**, didymin **(G,H)**, cardomin **(I,J)**, 2n **(K,L)**, morusin **(M,N)**, cyclomorusin **(O,P)**, morusinol **(Q,R)**, neocyclomorusin **(S,T)**, biochanin A **(U,V)**, and PC4 (4′-piperazinoacetophenone-CH_3_; **(W,X)** reference ligand: 2,2,4-trihydroxychalcone **(Y,Z)**.

The binding interactions of the representative compounds from each cluster with BACE1 were analyzed through molecular docking using AutoDock Vina and visualized using Discovery Studio Visualizer. The 12 compounds were **D3** (3′,7-diethoxy-5-hydroxy-4′-methoxyflavone), **3,5,7,3′,4′-pentamethoxyflavone, naringenin, didymin, cardomin, 2n** ((E)-3-(4-fluorophenyl)-1-(2-hydroxy-5-iodo-4-methoxyphenyl)prop-2-en-1-one), **morusin, cyclomorusin, morusinol**, neocyclomorusin, **biochanin A**, and **PC4**((2E)-3-(4-methylphenyl)-1-[4-(1-piperazinyl)phenyl]-2-propen-1-one). All these flavonoids were docked with the crystal structure of human BACE1 (pdb id: 2wjo) that was obtained from the PDB database, and 2,2,4-trihydroxychalcone was used as the reference ligand for this study. The results are shown in Figures 4A–Z and tabulated in Table 4.

**TABLE 4:** Result of the molecular docking of 12 compounds, which are representative of each cluster. Information includes the binding scores, number of hydrogen bond formed, residues involved in hydrogen bond, and others.

Cluster	Compound	Energy (kcal/mol)	No of H-bonds	H-bond interaction residue	Other interacting residue
1	3′,7-Diethoxy-5-hydroxy-4′-methoxyflavone (D3)	−8.10	2	Conventional hydrogen bond: Asn37	π–sigma: Val69
					π–π T-shaped: Phe108
					Alkyl: Ile126, Ile118, and Leu30
				Pi-donor hydrogen bond: Trp76	π–alkyl: Arg128, Tyr198, Phe108, and Trp115
					Unfavorable donor–donor: Ser36
2	3,5,7,3′,4′-Pentamethoxyflavone	−7.80	2	Conventional hydrogen bond: Trp76	π–π T-shaped: Phe108
				Carbon hydrogen bond: Ser35	Alkyl: Arg128, Val69, Leu30, Ile110, and Ile118
					π–alkyl: Val69, Trp115, and Trp76
3	Naringenin	−8.10	3	Conventional hydrogen bond: Gly34, Asp228, and Asp106	π–anion: Asp32
					π–π stacked: Tyr71
					π–π T-shaped: Phe108
4	Didymin	−9.80	6	Conventional hydrogen bond: Ile126, Trp76, and Asp228	π–sigma: Tyr71
				Carbon hydrogen bond: Gly230, Ser35, and Gly34	π–π T-shaped: Phe108
					π–alkyl: Leu30
5	Cardamonin	−7.10	3	Conventional hydrogen bond: Ile126, Asn37, and Trp76	π–alkyl: Val69 and Tyr71
6	(E)-3-(4-fluorophenyl)-1-(2-hydroxy-5-iodo-4-methoxyphenyl)prop-2-en-1-one (2n)	−7.90	1	Conventional hydrogen bond: Trp76	Halogen (fluorine): Lys107 and Gln73
					π–π T-shaped: Phe108
					Alkyl: Arg128
					π–alkyl: Tyr71 and Val69
7	Morusin	−9.40	1	Conventional hydrogen bond: Asp106	π–π stacked: Tyr71
					Alkyl: Leu30 and Ile118
					π–alkyl: Trp115 and Phe108
8	Cyclomorusin	−10.10	2	Conventional hydrogen bond: Asp106 and Asp228	π–π stacked: Tyr71
					π–π T-shaped: Phe108
					Alkyl: Leu30
9	Morusinol	−9.30	3	Conventional hydrogen bond: Asp106 and Gln73	Unfavorable acceptor–acceptor: Gly230
				Carbon hydrogen bond: Trp76	π–π stacked: Tyr71
10	Neocyclomorusin	−9.80	2	Carbon hydrogen bond: Trp76 and Ser35	π–anion: Asp32
					π–π stacked: Tyr71
11	Biochanin A	−8.70	3	Conventional hydrogen bond: Ser36, Asn37, and Trp76	π–π stacked: Tyr71
					π–π T-shaped: Phe108
					π–alkyl: Val69 and Arg128
12	(2E)-3-(4-Methylphenyl)-1-[4-(1-piperazinyl)phenyl]-2-propen-1-one (PC4)	−8.10	2	Conventional hydrogen bond: Ile126	Alkyl: Leu30
				Carbon hydrogen bond: Asn37	π–alkyl: Val69 and Leu30
13	Reference ligand: 2,2,4-trihydroxychalcone	−7.90	3	Conventional hydrogen bond: Asp106, Gln73, and Lys107	π–sigma: Val69
					π–π stacked: Tyr71
					π–π T-shaped: Phe108
					π–alkyl: Arg128

Based on Table 4, **cyclomorusin** (cluster 8) was predicted to bind to BACE1 with the lowest binding score, which is −10.10 kcal/mol. **Cyclomorusin** also has a lower binding score compared to the reference ligand (2,2,4-trihydroxychalcone), which was −7.90 kcal/mol. The hydroxyl group of **cyclomorusin** formed two hydrogen bonds with Asp106 (ring B) and also catalytic aspartic residues, Asp228 (ring A) of BACE1. All three rings of **cyclomorusin** also formed a π–π stacking interaction with Tyr71 of BACE1. Leu30 residue of BACE1 also interacts with the methyl group of the pentacyclic structure. However, the IC_50_ value of **cyclomorusin** was in contrast with the docking result, where **cyclomorusin** showed lower potency in inhibiting BACE1 compared to other flavonoids (101.2 μM).

Another compound, **neocyclomorusin**, which belongs to cluster 10, also showed a low binding score, which is −9.80 kcal/mol. Similar to **cylcomorusin**, **neocyclomorusin** showed lower potency in inhibiting BACE1 (146.1 μM) than the rest. Uniquely, these two compounds have a similar pentacyclic structure formed by the ring closure of the prenyl group. As can be seen in Figures 4S–T, the embedded oxygen in the pentacyclic structure of **neocyclomorusin** binds to Ser35 of BACE1 through a hydrogen bond. Moreover, the π–anion interaction was observed between ring B of **neocyclomorusin** and Asp32 of BACE1. This interaction of the π–anion is stronger than the hydrogen bond (Bartlett et al., 2013). The presence of these structures in **neocyclomorusin** may contribute to the lower binding energy against BACE1.

From all the representative clusters, **didymin** (cluster 4) had the highest number of hydrogen bonds, which was 6 (Ile126, Trp76, Asp228, Gly230, Ser35, and Gly34) compared to the reference ligand, where there were only three hydrogen bond interactions with Asp106, Gln73, and Lys107. **Didymin** showed a binding energy of −9.40 kcal/mol where hydroxyl groups from the two sugar molecules form the most hydrogen interactions with the amino acid residues of BACE1. One of the hydroxyl groups interacts with the catalytic aspartic residue, Asp228. Other interactions such as π–sigma (Tyr71), π–π T-shaped (Phe108), and π–alkyl (Leu30) were present between **didymin** and BACE1. Only the π–sigma interaction was observed between the residue of Tyr71 with the methyl group of the sugar moiety. Other π–π interactions involved the aromatic rings of **didymin**.

Compound **2n** ((E)-3-(4-fluorophenyl)-1-(2-hydroxy-5-iodo-4-methoxyphenyl)prop-2-en-1-one) is the only representative compound that has a halogen functional group. The fluorine group forms a van der Waals interaction with Lys107 and Gln73. Molecular docking results also showed that D3 (3′,7-diethoxy-5-hydroxy-4′-methoxyflavone), **Didymin**, **cardamonin**, **2n** ((E)-3-(4-fluorophenyl)-1-(2-hydroxy-5-iodo-4-methoxyphenyl)prop-2-en-1-one), **morusinol**, **neocyclomorusin**, and **biochanin A** share a common hydrogen bond interaction with Trp76. Moreover, all of the representative flavonoids including the reference ligand shared a common π–π interaction with Tyr71 except for **3,5,7,3′,4′-pentamethoxyflavone**.

## Discussion

Due to its favorable structures and properties, flavonoids have been considered a potential BACE1 inhibitor. However, to date, no flavonoids have advanced into clinical trials as BACE1 inhibitors. Here, an analysis of flavonoids that has been tested as BACE1 inhibitors from studies published from 2010 to 2022 was conducted to discover the pharmacophoric features of flavonoids against BACE1. In the clustering analysis, flavonoids were clustered based on their core fragments and binding interactions between flavonoids and BACE1 were analyzed using molecular docking. These two analyses were performed to determine the relationship between the chemical structure and inhibitory activity of flavonoids against BACE1. From the results of the clustering and molecular docking of 64 flavonoids compiled, several observations can be made.

First, compounds with the flavanone core fragments showed an apparent relationship between the 2D structure and bioactivity. Flavanone core fragments were divided into two clusters, where compounds in the two clusters differed by the presence of sugar moieties. The sugar moiety may increase inhibition toward BACE1, as seen in Table 3, where most of the flavonoids in cluster 4 have low IC_50_ values compared to those in cluster 3. This has also been discussed by Ali et al. (2019), where the number and position of sugar moieties and the different positions of the glycosidic linkages of flavanone may affect the inhibitory activity of BACE1. There are also a few studies that demonstrated that the presence of a sugar moiety in natural product compounds contributes to the inhibitory activity of BACE1 such as terpenoids isolated from *Dipsacus radix*, rubrofusarin, and derivatives from *Cassia obtusifolia* Linn (Shrestha et al., 2018; Wang et al., 2021).

The non-piperazine-substituted chalcone fragment also showed an obvious relationship between the 2D structure and bioactivity. Substitution at the *para* position of rings A and B with functional groups with decreasing electronegativity seems to result in an increase in the IC_50_ value. When ring A is substituted with a hydroxyl group at the *meta* position of ring A and there is no substitution on ring B, such as in the case of **cardamonin**, this produces the lowest IC_50_ value among the chalcones. Molecular docking shows that **cardamonin** formed three hydrogen bonds with BACE1 compared to only one by compound 2n ((E)-3-(4-fluorophenyl)-1-(2-hydroxy-5-iodo-4-methoxyphenyl)prop-2-en-1-one). In a study by Ma et al. (2011), a series of hydroxychalcones were evaluated for their inhibitory activities against BACE1. The structure–activity relationship from this study showed that the inhibitory activity of the chalcone against BACE1 was governed by the hydroxyl substituents on rings A and B of the chalcone, where the most potent chalcone was substituted with four hydroxyl groups (IC_50_ = 0.27 μM). The high potency may be attributed to the ability of the compounds to form hydrogen bonds with the catalytic site. Van der Waals interaction may also play an important part in the interaction between chalcone and BACE1. When compounds in cluster 6 were substituted at the *para* position in rings A and B with decreasing electronegativity (Br > Cl > CH_3_O and F > CH_3_O, respectively), the IC_50_ value increases. In addition, flavonoids with chalcone core fragments did not violate the “Lipinski’s rule,” which shows they have good absorption and bioavailability. Chalcone is also known as “privileged structures” as both natural and synthetic chalcone derivatives have shown compelling biological activities with clinical potential against different types of diseases (Zhuang et al., 2017).

Thirdly, the number and position of sugar moieties attached to the flavanone core play an important role in inhibiting BACE1. These sugar moieties can be linked to an aglycone as monosaccharides, disaccharides, or oligosaccharides. In this study, all flavanones in cluster 4 have different numbers of sugar moieties and positions attached to the core fragment. **Didymin**, which contains disaccharides, showed the most potent inhibitory activity against BACE1 compared to other flavanones in cluster 4. Moreover, the hydroxyl group from one of the sugar moieties in **didymin** interacts with catalytic aspartic residues of BACE1 (Asp228). Although **poncirin** also contains two disaccharides, it exhibited a different level of potency against BACE1 due to a different position of the glycosidic linkage. These results are supported by Shrestha et al. (2018), where the activity of rubrofusarin and its derivatives against AChE and BACE1 was investigated. The study found that the glucose moiety at position C6 of nor-rubrofusarin is responsible for the inhibitory activity against BACE1. Moreover, the presence of two sugar moieties inhibited AChE nine times more than a compound having no sugar moiety. Choi et al. (2016) also showed that sugar molecules of ginsenosides had the most interactions with the residues of the BACE1 active site through hydrogen bond and van der Waals interactions.

The majority of BACE1 inhibitors seem to have a large number of hydrogen bond donors and acceptors and form strong hydrogen bonds with Asp32 and Asp228 of BACE1 (Hernández et al., 2016). From this study, **didymin** has the most hydrogen bonds compared to other representative flavonoids and formed hydrogen bonds with Asp228. This is indicated by the low binding energy score (9.80 kcal/mol) and high inhibitory activity (IC_50_ = 2.34 μM) of **didymin**. This finding is supported by molecular docking studies that were performed to study the effects of flavonols and flavones as BACE1 inhibitors. In the BACE1 target interaction, myricetin was found to form the largest number of hydrogen bonds with BACE1. Two hydrogen bonds were formed with Asp32 through C3-OH of ring C. Several other hydrogen bonds were formed between Trp198 and C4′-OH and C5′-OH of ring B and between Gln73 and C7-OH of ring A (Shimmyo et al., 2008). A study by Shrestha et al. (2018) also showed that nor-rubrofusarin 6-O-β-D-glucoside had a binding energy of −8.34 kcal/mol in the allosteric inhibition mode with BACE1 and formed six hydrogen bonds with Gln303, Gln304, Glu339, and Gly156. In addition, the inhibition of BACE1 with nor-rubrofusarin 6-O-β-D-glucoside by catalytic inhibition showed a binding energy of -6.61 kcal/mol with six hydrogen bonds formed with Asp32, Trp76, Asn37, Ile26, and Tyr198 (Shrestha et al., 2018). Although **didymin** formed the most hydrogen bonds with BACE1, it violates two of “Lipinski’s rule,” as it has 14 hydrogen bond acceptors and 7 hydrogen bond donors due to the presence of the sugar moiety.

Another unique structure of flavonoid that needs to be highlighted is the pentacyclic structure that can be found in **cyclomorusin** and **neocyclomorusin**. The extra ring in both structures may contribute to the low binding energy score observed. However, previous study has reported that the pentacyclic structure of both compounds resulted in lower potency against BACE1, where a free hydroxyl group is needed for the BACE1 activity (Cho et al., 2011). Further study needs to be done on the pentacyclic structure to understand whether this structure significantly contributes to the BACE1 inhibitory activity. It should be noted that molecular dynamics stimulation would further enhance our understanding on the binding interaction between flavonoids and BACE1, which could not be performed in this study and should be performed in future studies. Molecular dynamics simulation provides more details on the movement of every atom in a protein compared to molecular docking, which only generates the binding mode of a ligand to a protein and predicts the number of possible conformations. In molecular dynamics simulation, it predicts important biomolecular processes such as conformational change, ligand binding, protein folding, and the position of all atoms at a very fine temporal resolution (Hollingsworth and Dror, 2018).

Despite various studies supporting flavonoids as potential BACE1 inhibitors, there are also significant challenges with the isolation, purification, and pharmacokinetic properties of the flavonoids. Flavonoids are very difficult to isolate, as only a small amount can be obtained at one time. Additionally, flavonoids are highly metabolized, with low solubility and poor oral absorption (Amawi et al., 2017). However, there are several approaches that could address these issues. In regard to its isolation, nano-harvesting has been applied to increase product yield. Kurepa et al. (2014) utilized nanoparticles such as anatase TiO_2_ to conjugate enediol and catechol group-rich flavonoids. This technique eliminates the use of organic solvents and yielded a higher percentage of flavonoid compounds (Kurepa et al., 2014). Moreover, structural modifications can be performed to increase the solubility and stability of flavonoids. The replacement of hydroxyl with an ethyl group in quercetin was shown to improve its stability against metabolic enzymes by preventing oxidative degradation. Insertion of the ethyl group also increases the lipophilicity of quercetin from 10.7% to 18.8% (Grande et al., 2016). Micro- and nano-delivery systems could also help to increase the bioavailability of flavonoid such as the use of nano-emulsions and nano-crystals (Amawi et al., 2017). Yi et al. (2017) developed a novel silybin nano-crystal using high-pressure homogenization. It was found that the release rate of silybin nano-crystal was faster *in vitro* and showed a higher peak concentration *in vivo* compared to the silybin coarse powder. This demonstrated that the nano-crystal technique increases bioavailability and is a promising oral drug delivery system for poorly soluble drugs such as flavonoids (Yi et al., 2017).

## Conclusion

In this study, an analysis was conducted on flavonoids that have been tested against BACE1 enzymes from 2010 to 2022. Specifically, the structure–activity relationship analysis was conducted, involving clustering and molecular docking. Several key findings from this study include: 1) flavanones with sugar moieties showed higher inhibitory activity than those without sugar moieties. Additionally, the number of sugar moieties and the position of glycosidic linkage affect the inhibitory activity. 2) Non-piperazine-substituted chalcones when substituted with functional groups with decreasing electronegativity at the *para* position of both rings result in a decreased inhibitory activity. Molecular docking indicates that ring A is involved in hydrogen bonding, whereas ring B is involved in van der Waals interaction with BACE1. 3) Hydrogen bond is an important interaction with the catalytic site of BACE1. It should be noted that the SAR analysis performed in this study is limited to 2D similarities only. Further studies involving other similarity metrics are warranted. Additionally, molecular dynamic studies are also warranted to study the behavior of BACE1 with flavonoids in full atomic detail and at very fine temporal resolution. Hence, these findings may aid in the design of highly potent and specific BACE1 inhibitors, which could aid in delaying the progression of AD.

## Data Availability

The original contributions presented in the study are included in the article/Supplementary Material; further inquiries can be directed to the corresponding author.

## References

[B1] Abeysinghe A. A. D. T. Deshapriya R. D. U. S. Udawatte C. (2020). Alzheimer's Disease; a Review of the Pathophysiological Basis and Therapeutic Interventions. Life Sci. 256, 117996. 10.1016/j.lfs.2020.117996 32585249

[B2] Ali M. Y. Jannat S. Edraki N. Das S. Chang W. K. Kim H. C. (2019). Flavanone Glycosides Inhibit β-site Amyloid Precursor Protein Cleaving Enzyme 1 and Cholinesterase and Reduce Aβ Aggregation in the Amyloidogenic Pathway. Chemico-Biological Interact. 309, 108707. 10.1016/j.cbi.2019.06.020 31194956

[B3] Amawi H. Ashby C. R. Tiwari A. K. (2017). Cancer Chemoprevention through Dietary Flavonoids: What's Limiting? Chin. J. Cancer 36, 1–13. 10.1186/s40880-017-0217-4 28629389 PMC5477375

[B4] Ashrafian H. Zadeh E. H. Khan R. H. (2021). Review on Alzheimer's Disease: Inhibition of Amyloid Beta and Tau Tangle Formation. Int. J. Biol. Macromol. 167, 382–394. 10.1016/j.ijbiomac.2020.11.192 33278431

[B5] Babiaka S. B. Nia R. Abuga K. O. Mbah J. A. Nziko V. d. P. N. Paper D. H. (2020). Antioxidant Potential of Flavonoid Glycosides from *Manniophyton Fulvum* Müll. (Euphorbiaceae): Identification and Molecular Modeling. Sci. Afr. 8, e00423. 10.1016/j.sciaf.2020.e00423

[B6] Banks W. A. (2009). Characteristics of Compounds that Cross the Blood-Brain Barrier. BMC Neurol. 9 (1), S3–S5. 10.1186/1471-2377-9-S1-S3 19534732 PMC2697631

[B7] Bartlett G. J. Newberry R. W. Vanveller B. Raines R. T. Woolfson D. N. (2013). Interplay of Hydrogen Bonds and N→π* Interactions in Proteins. J. Am. Chem. Soc. 135 (49), 18682–18688. 10.1021/ja4106122 24256417 PMC3940535

[B8] Carecho R. Carregosa D. Dos Santos C. N. (2021). Low Molecular Weight (poly)Phenol Metabolites across the Blood-Brain Barrier: The Underexplored Journey. Brain. plast. 6 (2), 193–214. 10.3233/BPL-200099 33782650 PMC7990460

[B9] Chakraborty S. Kumar S. Basu S. (2011). Conformational Transition in the Substrate Binding Domain of β-secretase Exploited by NMA and its Implication in Inhibitor Recognition: BACE1-Myricetin a Case Study. Neurochem. Int. 58 (8), 914–923. 10.1016/j.neuint.2011.02.021 21354237

[B10] Chen G.-f. Xu T.-h. Yan Y. Zhou Y.-r. Jiang Y. Melcher K. (2017). Amyloid Beta: Structure, Biology and Structure-Based Therapeutic Development. Acta Pharmacol. Sin. 38 (9), 1205–1235. 10.1038/aps.2017.28 28713158 PMC5589967

[B11] Cho J. K. Ryu Y. B. Curtis-Long M. J. Kim J. Y. Kim D. Lee S. (2011). Inhibition and Structural Reliability of Prenylated Flavones from the Stem Bark of *Morus Lhou* on β-secretase (BACE-1). Bioorg. Med. Chem. Lett. 21 (10), 2945–2948. 10.1016/j.bmcl.2011.03.060 21511472

[B12] Choi R. J. Roy A. Jung H. J. Ali M. Y. Min B.-S. Park C. H. (2016). BACE1 Molecular Docking and Anti-alzheimer's Disease Activities of Ginsenosides. J. Ethnopharmacol. 190, 219–230. 10.1016/j.jep.2016.06.013 27275774

[B13] Coimbra J. R. M. Marques D. F. F. Baptista S. J. Pereira C. M. F. Moreira P. I. Dinis T. C. P. (2018). Highlights in BACE1 Inhibitors for Alzheimer's Disease Treatment. Front. Chem. 6, 178. 10.3389/fchem.2018.00178 29881722 PMC5977085

[B14] Cummings J. L. Goldman D. P. Simmons‐Stern N. R. Ponton E. (2021). The Costs of Developing Treatments for Alzheimer's Disease: A Retrospective Exploration. Alzheimer's Dementia 18 (3), 469–477. 10.1002/alz.12450 PMC8940715 34581499

[B15] Dallakyan S. Olson A. J. (2015). Small-Molecule Library Screening by Docking with Pyrx. Biol. Methods Protoc. 1263, 243–250. 10.1007/978-1-4939-2269-7_19 25618350

[B16] Feigin V. L. Nichols E. Alam T. Bannick M. S. Beghi E. Blake N. (2019). Global, Regional, and National Burden of Neurological Disorders, 1990-2016: a Systematic Analysis for the Global Burden of Disease Study 2016. Lancet Neurol. 18 (5), 459–480. 10.1016/S1474-4422(18)30499-X 30879893 PMC6459001

[B17] Grande F. Parisi O. I. Mordocco R. A. Rocca C. Puoci F. Scrivano L. (2016). Quercetin Derivatives as Novel Antihypertensive Agents: Synthesis and Physiological Characterization. Eur. J. Pharm. Sci. 82, 161–170. 10.1016/j.ejps.2015.11.021 26631584

[B18] Hampel H. Caraci F. Cuello A. C. Caruso G. Nisticò R. Corbo M. (2020). A Path toward Precision Medicine for Neuroinflammatory Mechanisms in Alzheimer's Disease. Front. Immunol. 11, 456. 10.3389/fimmu.2020.00456 32296418 PMC7137904

[B19] Hampel H. Vassar R. De Strooper B. Hardy J. Willem M. Singh N. (2021). The β-Secretase BACE1 in Alzheimer's Disease. Biol. Psychiatry 89 (8), 745–756. 10.1016/j.biopsych.2020.02.001 32223911 PMC7533042

[B20] Han J. Ji Y. Youn K. Lim G. Lee J. Kim D. H. (2019). Baicalein as a Potential Inhibitor against BACE1 and AChE: Mechanistic Comprehension through *In Vitro* and Computational Approaches. Nutrients 11 (11), 2694. 10.3390/nu11112694 PMC6893645 31703329

[B21] Hernández-Rodríguez M. Correa-Basurto J. Gutiérrez A. Vitorica J. Rosales-Hernández M. C. (2016). Asp32 and Asp228 Determine the Selective Inhibition of BACE1 as Shown by Docking and Molecular Dynamics Simulations. Eur. J. Med. Chem. 124, 1142–1154. 10.1016/j.ejmech.2016.08.028 27639619

[B22] Hollingsworth S. A. Dror R. O. (2018). Molecular Dynamics Simulation for All. Neuron 99 (6), 1129–1143. 10.1016/j.neuron.2018.08.011 30236283 PMC6209097

[B23] Huang L.-K. Chao S.-P. Hu C.-J. (2020). Clinical Trials of New Drugs for Alzheimer Disease. J. Biomed. Sci. 27 (1), 1–13. 10.1186/s12929-019-0609-7 31906949 PMC6943903

[B24] Khan M. T. H. Orhan I. Şenol F. S. Kartal M. Şener B. Dvorská M. (2009). Cholinesterase Inhibitory Activities of Some Flavonoid Derivatives and Chosen Xanthone and Their Molecular Docking Studies. Chemico-Biological Interact. 181 (3), 383–389. 10.1016/j.cbi.2009.06.024 19596285

[B25] Kratz J. M. Grienke U. Scheel O. Mann S. A. Rollinger J. M. (2017). Natural Products Modulating the HERG Channel: Heartaches and Hope. Nat. Prod. Rep. 34 (8), 957–980. 10.1039/C7NP00014F 28497823 PMC5708533

[B26] Kurepa J. Nakabayashi R. Paunesku T. Suzuki M. Saito K. Woloschak G. E. (2014). Direct Isolation of Flavonoids from Plants Using Ultra‐small Anatase TiO 2 Nanoparticles. Plant J. 77 (3), 443–453. 10.1111/tpj.12361 24147867 PMC3935720

[B27] Lee S. Youn K. Lim G. Lee J. Jun M. (2018). *In Silico* docking and *In Vitro* Approaches towards BACE1 and Cholinesterases Inhibitory Effect of Citrus Flavanones. Molecules 23 (7), 1509. 10.3390/molecules23071509 PMC6100189 29932100

[B28] Ma L. Yang Z. Li C. Zhu Z. Shen X. Hu L. (2011). Design, Synthesis and SAR Study of Hydroxychalcone Inhibitors of Human β-secretase (BACE1). J. Enzyme Inhibition Med. Chem. 26 (5), 643–648. 10.3109/14756366.2010.543420 21222511

[B29] Malone K. Hancox J. C. (2020). QT interval prolongation and *Torsades de Pointes* with donepezil, rivastigmine and galantamine. Ther. Adv. Drug Saf. 11, 204209862094241. 10.1177/2042098620942416 PMC7436781 32874532

[B30] Mathew B. Oh J. M. Baty R. S. Batiha G. E.-S. Parambi D. G. T. Gambacorta N. (2021). Piperazine-substituted Chalcones: A New Class of MAO-B, AChE, and BACE-1 Inhibitors for the Treatment of Neurological Disorders. Environ. Sci. Pollut. Res. 28 (29), 38855–38866. 10.1007/s11356-021-13320-y PMC7980107 33743158

[B31] Mehta D. Jackson R. Paul G. Shi J. Sabbagh M. (2017). Why Do Trials for Alzheimer's Disease Drugs Keep Failing? A Discontinued Drug Perspective for 2010-2015. Expert Opin. Investigational Drugs 26 (66), 735–739. 10.1080/13543784.2017.1323868 PMC5576861 28460541

[B32] Mphahlele M. Agbo E. Gildenhuys S. (2018). Synthesis and Evaluation of the 4-Substituted 2-Hydroxy-5-Iodochalcones and Their 7-Substituted 6-Iodoflavonol Derivatives for Inhibitory Effect on Cholinesterases and β-Secretase. Int. J. Mol. Sci. 19 (12), 4112. 10.3390/ijms19124112 PMC6321475 30567381

[B33] Nazarko L. (2019). Dementia 2. Alzheimer's Disease: Diagnosis, Treatment and Managment. Br. J. Healthc. Assistants 13 (7), 329–335. 10.12968/bjha.2019.13.7.329

[B34] Panche A. N. Diwan A. D. Chandra S. R. (2016). Flavonoids: an Overview. J. Nutr. Sci. 5, 1–15. 10.1017/jns.2016.41 PMC5465813 28620474

[B35] Pardridge W. M. (2005). The Blood-Brain Barrier: Bottleneck in Brain Drug Development. Neurotherapeutics 2 (1), 3–14. 10.1602/neurorx.2.1.3 PMC539316 15717053

[B36] Plaingam W. Sangsuthum S. Angkhasirisap W. Tencomnao T. (2017). *Kaempferia Parviflora* Rhizome Extract and *Myristica Fragrans* Volatile Oil Increase the Levels of Monoamine Neurotransmitters and Impact the Proteomic Profiles in the Rat hippocampus: Mechanistic Insights into Their Neuroprotective Effects. J. Traditional Complementary Med. 7 (4), 538–552. 10.1016/j.jtcme.2017.01.002 PMC5634759 29034205

[B37] Reis J. Gaspar A. Milhazes N. Borges F. (2017). Chromone as a Privileged Scaffold in Drug Discovery: Recent Advances. J. Med. Chem. 60 (19), 7941–7957. 10.1021/acs.jmedchem.6b01720 28537720

[B38] Shimmyo Y. Kihara T. Akaike A. Niidome T. Sugimoto H. (2008). Flavonols and Flavones as BACE-1 Inhibitors: Structure-Activity Relationship in Cell-free, Cell-Based and In Silico Studies Reveal Novel Pharmacophore Features. Biochimica Biophysica Acta (BBA) - General Subj. 1780 (5), 819–825. 10.1016/j.bbagen.2008.01.017 18295609

[B39] Shrestha S. Seong S. Paudel P. Jung H. Choi J. (2018). Structure Related Inhibition of Enzyme Systems in Cholinesterases and BACE1 *In Vitro* by Naturally Occurring Naphthopyrone and its Glycosides Isolated from Cassia Obtusifolia. Cassia obtusifoliaMolecules 23 (1), 69. 10.3390/molecules23010069 PMC6017707 29283428

[B40] Stieger B. Mahdi Z. M. Jäger W. (2017). Intestinal and Hepatocellular Transporters: Therapeutic Effects and Drug Interactions of Herbal Supplements. Annu. Rev. Pharmacol. Toxicol. 57, 399–416. 10.1146/annurev-pharmtox-010716-105010 27648763

[B41] Ting Chan X. L. Xu C. Ng W. Zhu L. Zhou F. (2014). The Interactions of Herbal Compounds with Human Organic Anion/Cation Transporters. J. Pharmacogenomics Pharmacoproteomics 05 (5), 142. 10.4172/2153-0645.1000142

[B42] Tran T.-S. Tran T.-D. Tran T.-H. Mai T.-T. Nguyen N.-L. Thai K.-M. (2020). Synthesis, In Silico and *In Vitro* Evaluation of Some Flavone Derivatives for Acetylcholinesterase and BACE-1 Inhibitory Activity. Molecules 25 (18), 4064. 10.3390/molecules25184064 PMC7570966 32899576

[B43] Trott O. Olson A. J. (2009). Autodock Vina: Improving the Speed and Accuracy of Docking with A New Scoring Function, Efficient Optimization, and Multithreading. J. Comput. Chem. 31 (2), 21334. 10.1002/jcc.21334 PMC3041641 19499576

[B44] Vauzour D. Vafeiadou K. Rodriguez-Mateos A. Rendeiro C. Spencer J. P. E. (2008). The Neuroprotective Potential of Flavonoids: a Multiplicity of Effects. Genes Nutr. 3 (3-4), 115–126. 10.1007/s12263-008-0091-4 18937002 PMC2593006

[B45] Wagle A. Seong S. H. Shrestha S. Jung H. A. Choi J. S. (2019). Korean Thistle (Cirsium Japonicum Var. Maackii (Maxim.) Matsum.): A Potential Dietary Supplement against Diabetes and Alzheimer's Disease. Molecules 24 (3), 649. 10.3390/molecules24030649 PMC6385118 30759846

[B46] Walsh S. Merrick R. Milne R. Brayne C. (2021). Aducanumab for Alzheimer's Disease? BMJ 374, n1682. 10.1136/bmj.n1682 34226181 PMC8258645

[B47] Wang Z. Y. Zhang X. D. Whang W. K. (2021). The Effect of Terpenoids of Dipsacus Asperoides against Alzheimer's Disease and Development of Simultaneous Analysis by High Performance Liquid Chromatography. Nat. Product. Commun. 16 (9), 1934578X2110446–11. 10.1177/1934578X211044603

[B48] Williamson G. Kay C. D. Crozier A. (2018). The Bioavailability, Transport, and Bioactivity of Dietary Flavonoids: A Review from a Historical Perspective. Compr. Rev. Food Sci. Food Saf. 17 (5), 1054–1112. 10.1111/1541-4337.12351 33350159

[B49] Xu Y. Li M.-j. Greenblatt H. Chen W. Paz A. Dym O. (2012). Flexibility of the Flap in the Active Site of BACE1 as Revealed by Crystal Structures and Molecular Dynamics Simulations. Acta Crystallogr. D. Biol. Cryst. 68 (1), 13–25. 10.1107/S0907444911047251 22194329

[B50] Yi T. Liu C. Zhang J. Wang F. Wang J. Zhang J. (2017). A New Drug Nanocrystal Self-Stabilized Pickering Emulsion for Oral Delivery of Silybin. Eur. J. Pharm. Sci. 96, 420–427. 10.1016/j.ejps.2016.08.047 27575878

[B51] Youn K. Jun M. (2019). Biological Evaluation and Docking Analysis of Potent BACE1 Inhibitors from Boesenbergia Rotunda. Boesenb. rotundaNutrients 11 (3), 662. 10.3390/nu11030662 PMC6471523 30893825

[B52] Youn K. Lee J. Ho C.-T. Jun M. (2016a). Discovery of Polymethoxyflavones from Black Ginger ( Kaempferia Parviflora ) as Potential β-secretase (BACE1) Inhibitors. J. Funct. Foods 20, 567–574. 10.1016/j.jff.2015.10.036

[B53] Youn K. Park J.-H. Lee J. Jeong W.-S. Ho C.-T. Jun M. (2016b). The Identification of Biochanin A as a Potent and Selective β-Site App-Cleaving Enzyme 1 (Bace1) Inhibitor. Nutrients 8 (10), 637. 10.3390/nu8100637 PMC5084024 27754406

[B54] Youn K. Yu Y. Lee J. Jeong W.-S. Ho C.-T. Jun M. (2017). Polymethoxyflavones: Novel β-Secretase (BACE1) Inhibitors from Citrus Peels. Nutrients 9 (9), 973. 10.3390/nu9090973 PMC5622733 28869548

[B55] Zhuang C. Zhang W. Sheng C. Zhang W. Xing C. Miao Z. (2017). Chalcone: a Privileged Structure in Medicinal Chemistry. Chem. Rev. 117 (12), 7762–7810. 10.1021/acs.chemrev.7b00020 28488435 PMC6131713

[B56] Zhumanova K. Lee G. Baiseitova A. Shah A. B. Kim J. H. Kim J. Y. (2021). Inhibitory Mechanism of O-Methylated Quercetins, Highly Potent β-secretase Inhibitors Isolated from Caragana Balchaschensis (Kom.) Pojark. J. Ethnopharmacol. 272, 113935. 10.1016/j.jep.2021.113935 33609726

